# 

**Published:** 1901-01

**Authors:** 


### LIST OF CONTRIBUTORS TO VOLUME XXII.

Allan, Dr. George S.
Anderson, Martha, M.D.
Andrews, R. R., A.M., D.D.S.
Arrington, Dr. B. F.
Bascom, Dr. H. S.
Benton, Dr. J. H.
Bierworth, J. C., M.D.
Bogue, Dr. E. A.
Bogue, Frederick L., M.D., D.D.S.
Brockway, Dr. A. H.
Carpenter, George T., M.D.
Carr, Dr. William.
Chilcott, L. S., D.D.S.
Chittenden, Charles C., D.D.S.
Danforth, Guy R., D.D.S.
Davenport, Dr. A. F.
Decker, W. E., D.D.S.
Eames, George F., M.D.
Ellerbeck, William Leon, D.D.S.
Fletcher, M. H., D.D.S., M.D., M.S.
Hamilton, Harry F., D.M.D.
Hatch, Henry D., D.D.S.
Heyke, Dr. John E.
Hunter, Dr. R. W.
Johnson, Supt. G. E.
Kiernan, James G., M.D.
Latham, Vida A., M.D., D.D.S., F.R.M.S.
Libbey, J. A., D.D.S.
Maxfield, George A., D.D.S.
Mead, G. N. P., M.D.
Mills, Dr. G. Alden.
Musson, Emma E., M.D.
Nichol, James P., D.D.S.
Onderdonk, Dr. T. W.
Parmele, Dr. George L.
Pierce, C. N., D.D.S.
Shumway, T. D.
Smith, B. Holly, M.D., D.D.S.
Smith, D. D., D.D.S., M.D.
Steeves, Alice M., D.D.S.
Talbot, Eugene S., M.D., D.D.S.
Thomas, Frank McS.
Thorpe, Dr. Burton L.
Turner, Dr. V. E.
Watkins, J. Conard, D.D.S.
Wilson, Erastus, M.D.
Wright, C. M., D.D.S.

